# Design, Implementation, and Examination of a Remote Patient Monitoring System for Pediatric Obesity: Protocol for an Open Trial Pilot Study

**DOI:** 10.2196/29858

**Published:** 2021-07-28

**Authors:** Crystal Lim, Laura Rutledge, Shanda Sandridge, Krista King, Darryl Jefferson, Tanya Tucker

**Affiliations:** 1 Department of Psychiatry & Human Behavior University of Mississippi Medical Center Jackson, MS United States; 2 Department of Pediatrics University of Mississippi Medical Center Jackson, MS United States; 3 Center for Telehealth University of Mississippi Medical Center Jackson, MS United States

**Keywords:** digital health, eHealth, obesity, pediatric obesity, pediatrics, remote patient monitoring, telemedicine, weight management

## Abstract

**Background:**

Pediatric obesity is a critical public health issue. Augmenting care in multidisciplinary pediatric obesity clinics with innovative evidence-based technology to improve weight status and health outcomes is needed.

**Objective:**

This study describes the design and methods of an open trial pilot study to examine a remote patient monitoring system (RPMS) for children aged 8-17 years who are receiving treatment in a multidisciplinary pediatric obesity clinic.

**Methods:**

Participants will include 45 youth with obesity and their parents. Families will receive standard care in the clinic and the RPMS for 3 months. The RPMS consists of a tablet, weight scale, and pedometer. The system provides daily educational content and involves the use of the pedometer and weekly weigh-ins. Children and parents will complete baseline, posttreatment (month 3), and follow-up assessments (month 6). The primary aim of the study is to examine feasibility and satisfaction with the RPMS and assess its initial effectiveness.

**Results:**

We hypothesize high feasibility and satisfaction, with rates over 75%. Furthermore, after RPMS treatment, children will exhibit improved weight status, health outcomes, dietary intake, physical activity, health-related quality of life, self-efficacy, and home-food environment compared to before treatment. These gains are expected to persist at follow-up.

**Conclusions:**

This study is novel in that it is the first to design, implement, and examine an RPMS in a pediatric obesity clinic. If the RPMS is feasible, effective, and easily accessible, it may prove to be a practical, acceptable, and cost-effective weight management treatment for youth seeking treatment for severe obesity.

**Trial Registration:**

ClinicalTrials.gov NCT04029597; https://clinicaltrials.gov/ct2/show/NCT04029597

**International Registered Report Identifier (IRRID):**

DERR1-10.2196/29858

## Introduction

Pediatric obesity is a critical public health issue in the United States. Serious comorbid health conditions including type 2 diabetes, heart disease, stroke, and cancers are common [1]. Younger individuals with obesity burden the health care system [2]. Specifically, pediatric obesity accounts for health care costs of over US $14 billion annually in the United States [3]. Efficacious pediatric obesity interventions are necessary to mitigate long-term health consequences and reduce health costs.

Standard care in multidisciplinary pediatric obesity clinics decrease adiposity in approximately 50% of treated youth [4,5]. However, providing and maintaining consistent care can be challenging when working with underserved children and families [6,7]. In Mississippi, over 50% of the state is rural, more than 35% of the population is Black or African American, and almost one-third of children live in poverty [8]. Mississippi also has the highest rates of pediatric obesity in the United States [9]. There is a significant need for innovative evidence-based technology to improve health and weight status.

Telemedicine use has increased owing to high rates of computer ownership, tablets, and cellphones, as well as easier internet access [10]. Insurance companies and health care organizations use telemedicine to support chronic disease management by developing remote patient monitoring systems (RPMSs) [10]. RPMSs provide patients with hardware and software that medically monitor health outcomes, provide educational information, automatically analyze health data, and alert patients and care providers of concerns [10]. RPMS efficacy studies have demonstrated improved health status in adults with chronic health conditions [11], such as diabetes [12], congestive heart failure [13], and obesity [14]. Nonetheless, applications of RPMSs in pediatric populations are limited. Four published studies have examined the applications of RPMSs in youth, having particularly focused on those with sleep apnea [15], cardiovascular implants [16], cancer [17], and type 1 diabetes [18]. These studies demonstrated feasibility, effectiveness, and reduced health care costs in pediatric populations; however, RPMSs have not been integrated into pediatric obesity clinics.

The primary objective of this study is to describe the design and methods of an open trial to test an RPMS designed to provide supplemental care to youth receiving treatment in a pediatric weight management clinic in Mississippi.

## Methods

### Ethics Approval and Trial Registration

This study was approved by the institutional review board at the University of Mississippi Medical Center (protocol# 2017-0083) and is registered on ClinicalTrials.gov (protocol# NCT04029597).

### Study Aims

The primary objective of this open trial is to pilot test an RPMS designed to provide supplemental health care to youth who are receiving specialty medical care in a multidisciplinary pediatric obesity clinic. Specifically, we will assess initial feasibility and satisfaction and examine initial effectiveness of the RPMS for 3 months. We hypothesize high feasibility with the use of the RPMS and satisfaction rates over 75%, and after treatment children are expected to exhibit improved health outcomes compared to before treatment and their parents are expected to report improvements in their children’s health-related quality of life and home-food environment.

### Study Design

This study will utilize an open trial design to examine the initial feasibility and effectiveness of implementing an RPMS in a pediatric obesity clinic. Participating families will receive standard care and the RPMS. After providing consent and assent, children and parents will complete questionnaires and objective measures as part of the pretreatment assessment. After using the RPMS for 3 months, families will complete the posttreatment assessment during a regularly scheduled follow-up appointment with health care providers at the pediatric weight management clinic. After an additional 3 months, participating families will also complete a follow-up assessment, similar to the pre- and posttreatment assessments, during a regularly scheduled clinic visit.

### Study Setting

The study will be conducted at the University of Mississippi Medical Center (UMMC) Pediatric Weight Management Clinic, a multidisciplinary outpatient medical clinic designed to treat children with obesity and related medical comorbidities. The clinic providers include a pediatric nurse practitioner and pediatric dietician who see every patient, as well as a clinical psychologist and mental health specialists who assess and treat patients with suspected mental health concerns. The study is being conducted in partnership with the UMMC Center for Telehealth, who assisted with the development of the RPMS intervention materials and provides oversight regarding RPMS equipment and participants’ use and interaction with the RPMS.

### Participants

The study will recruit 45 families with a child aged 8-17 years who attends an outpatient pediatric obesity clinic and has a weight status in the obese range (BMI equal to or above the 95th percentile for age and gender), and parents and children who are fluent in English. Exclusion criteria are the following: (1) the child or parent having a history of cognitive impairment (developmental delay or intellectual disability) reported by the parent, which would impact their ability to understand and complete questionnaires or interact with the RPMS; and (2) the child having a medical condition, reported by parents, which may prohibit wearing of the actigraph device (eg, pacemaker).

Potentially eligible participants will be recruited through the pediatric weight management outpatient multidisciplinary clinic. When families present for a scheduled appointment, providers will provide them information about the study and ask if they are interested in knowing more about it. A trained research team member will discuss the study in more detail and obtain child assent, parent permission, and parent consent to participate. The research assistant will then assist the child and parent with the completion of baseline questionnaires and assessments.

### Remote Patient Monitoring

The RPMS was developed in collaboration with UMMC Center of Telehealth. Patients enrolled in this open trial of the RPMS will interact with the system on a daily basis and with UMMC Center for Telehealth nurse care coordinators and research and clinical staff on an as-needed basis. The RPMS consists of an electronic tablet, weight scale, and pedometer. Patients will be asked to wear the pedometer daily to track engagement in physical activity and weigh themselves weekly to track weight during the 3-month period. Educational information specific to pediatric obesity–related medical complications and healthy eating and engagement in physical activity will be presented daily through brief presentations and video clips. Educational material and information were developed on the basis of behavioral family weight management treatments for pediatric obesity [19,20] and the social cognitive theory [21]. Daily assessments, which will include questions regarding health status and health behaviors, will be self-reported by the patient through the software program on the electronic tablet. Responses and vital signs and symptoms will be monitored by nurse care coordinators. The program contains a care management platform software that allows for data trends to be developed and analyzed to identify at-risk patients requiring action, such as follow-up phone calls to patients from the nurse coordinator and nurse coordinator informing the research and clinical team about patient symptoms. Solutions discussed and provided by the nurse coordinator and clinical research team may consist of the following: personalized interventions, targeted education, health coaching, behavior modification, medication adherence, and content to motivate patients. All health sessions are designed for patient engagement by providing quality educational content and self-management skills. The UMMC Center for Telehealth RPMS is linked to patients’ electronic medical record (ie, Epic) to facilitate communication between the nurse care coordinators and the medical team at the pediatric obesity. In addition, if patients have questions or need to contact the medical team the nurse care coordinators can facilitate that process, but patients and families are also able to contact (via secure messages, call, etc) the medical team providers, consistent with standard clinic procedures.

### Data Collection and Measures

Children and parents will complete the following measures and assessments at pretreatment (baseline), posttreatment (month 3), and follow-up (month 6) unless otherwise specified.

#### Child-Completed Questionnaires and Assessments

##### Height and Weight

Height without shoes will be measured using a stadiometer. Weight will be measured using a certified digital scale with 1 layer of clothing on and without shoes. Measurements will be conducted by trained clinical staff or research personnel. Data will be used to calculate child BMI, BMI *z* scores, and BMI percentiles using age- and gender-specific norms.

##### Health Outcomes

Standard procedures in the pediatric obesity clinic will be used by trained nursing or medical professionals to measure resting heart rate and blood pressure of children. In addition, results from routine blood tests assessing glucose and hemoglobin A_1c_ levels will be obtained from the child’s medical records.

##### Dietary Intake

Children will complete a 24-hour dietary recall by using the Automated Self-administered 24-hour Recall (ASA24) system. ASA24 is a free, secure web-based tool developed by the National Cancer Institute for research use. The ASA24-2018 was developed for children and adults aged 10 years and older and guides participants through the completion of a 24-hour dietary recall by asking about food intake and estimated portion sizes during the previous day. Responses are scored using the US Department of Agriculture’s Food and Nutrient Database for Dietary Studies.

##### Physical Activity

Children will wear an ActiGraph GT3X+ Activity Monitor on their waist for 24 hours a day for 1 week. The ActiGraph GT3X + Activity Monitor [22] measures child energy expenditure. After wearing the device for 7 days, families will return the actigraph via a self-addressed stamped envelope provided by research personnel at each assessment time point.

##### Self-Efficacy

To assess self-efficacy for eating healthier and engaging in physical activity, children will complete the Child Dietary Self-Efficacy Scale [23], which consists of 15 items that children answer using a 3-point Likert scale. Children will also complete the Self-Efficacy for Physical Activity Scale [24], which consists of 5 items specifically regarding the management of physical activity–related barriers. Both measures have been used previously in pediatric research and have acceptable reliability and validity.

##### Health-Related Quality of Life

To assess child quality of life, the Pediatric Quality of Life Inventory (PedsQL) [25] will be utilized. The PedsQL consists of 23 items and has been utilized with a variety of pediatric populations, including obesity, and it has good reliability and validity.

##### Treatment Satisfaction

Upon posttreatment assessment, children will complete the treatment satisfaction questionnaire, which was developed for this study and based on a previous study [26]. The questionnaire asks children to rate how much they agree or disagree with 10 statements.

#### Parent-Completed Questionnaires and Assessments

##### Demographic and Health History Information

At the baseline assessment, parents will complete a demographic questionnaire to obtain information about the child and parent. In addition, parents will answer questions about their child’s and their own medical history. Parents will also be asked to provide their contact information, specifically postal address, phone numbers, and email addresses and the name and phone number of another family member the research team can contact if we are unable to contact the parent.

##### Height and Weight

Parent height and weight will be measured using the same aforementioned methods used for children. Measurements will be used to calculate parent BMI and determine parent weight status.

##### Home-Food Environment

The Home Food Inventory [27] will be completed by parents to assess the availability of healthy and unhealthy food at home. The measure was developed for use in health promotion research and has demonstrated adequate reliability and validity.

##### Child Health-Related Quality of Life

Parents will complete the parent-proxy form of the PedsQL [25] to provide perceptions of their children’s quality of life. The parent-proxy form of the PedsQL has demonstrated good reliability and validity.

##### Treatment Satisfaction

Parents will also complete the treatment satisfaction questionnaire developed for this study upon posttreatment assessment [26]. The questionnaire asks parents to rate how much they agree or disagree with 12 statements about the RPMS.

#### Compensation

Families will receive compensation for completing each assessment. Total compensation for participating in the study will be US $90 during the 6-month study period.

### Statistical Analyses

To examine aim 1, descriptive statistics (including mean [SD] and percentage values) of engagement with the RPMS will be used to examine feasibility and satisfaction scores on the basis of child and parent reports completed during the posttreatment assessment. To examine aim 2—initial effectiveness of the RPMS—repeated-measures analysis of variance will be conducted to determine whether there are significant differences in outcomes over time. Two-sample *t* tests will be used to compare differences in outcomes from pre- to posttreatment, pretreatment to follow-up, and posttreatment to follow-up. If necessary (eg, outcome data are skewed), nonparametric tests will be used to compare pretreatment, posttreatment, and follow-up scores. Maximum likelihood techniques will be utilized for missing data to provide unbiased and accurate estimates of variables regardless of the pattern of missing data (ie, random or completely random). Statistical significance will be set at *P*<.05.

## Results

The study received ethics approval from the institutional review board in January 2019. Recruitment and enrollment began in August 2019. As of April 2021, 42 youth and their caregivers were enrolled in the study; however, participants continue to receive the RPMS intervention, and posttreatment and follow-up assessments are underway. Future outcomes will be published in professional peer-reviewed health-related research journals and presented at national, regional, or state-level professional meetings and conferences. Preliminary findings will be used to inform the development of a future study that will include a randomized controlled trial containing a larger sample of families and a standard care control group, evaluation of a refined RPMS, and cost-effectiveness analyses.

## Discussion

Here we describe the rationale and design of an open trial examining the feasibility, satisfaction, and effectiveness of an RPMS for youth receiving multidisciplinary treatment for obesity. There is a need to develop and implement treatments that complement clinical treatment, which are feasible, accessible, and useful for families from rural and underserved areas, who have obesity and related comorbidities. There is a lack of accessible treatments that address severe pediatric obesity. Bariatric surgery is effective [28,29] but is not currently available for youth in our state; hence, the implementation of more intensive treatments is necessary to address morbid pediatric obesity for our patients.

The RPMS is novel in its use of telehealth technology, connection with the electronic medical record, and easier access to health care information and health care providers for patients and their families. The RPMS developed in this study provides daily education and support for implementing lifestyle changes among individuals with severe obesity, who need more intensive approaches than those that are available in outpatient clinics and are constrained by time and space demands. Outpatient pediatric weight management clinics are typically not able to provide the amount of treatment recommended by the US Preventative Task Force, which is 26 hours of treatment in a year [30]. Thus, a feasible, appealing, and effective web-based intervention, such as our RPMS, may serve as a suitable method for pediatric obesity clinics to provide the recommended amount of treatment.

It is important to identify potential limitations of the current study and potential directions for future studies. First, the sample size for this pilot open trial design is relatively small. However, the proposed sample size is larger than that in previous studies on pediatric RPMSs [18]. If feasibility, satisfaction, and effectiveness data are promising, the research team intends to pursue additional grant funding to expand the sample size and use a randomized design to continue to evaluate the feasibility and effectiveness of the RPMS in this population. Second, the RPMS being evaluated here was designed by the pediatric obesity clinic providers at this specific clinic for its specific target population. Generally, among the patients treated at this clinic, approximately 60% are African American or Black, and approximately 35% live in rural areas of the state [31]. Thus, this specific RPMS may not be generalizable to clinics located in other parts of the country. Third, the length of treatment with the RPMS is 3 months. Though the treatment is intensive (ie, every day), it may not provide sufficient time to result in long-term behavioral changes.

This open trial will examine the initial feasibility, acceptability, and effectiveness of the RPMS for racially diverse youth with severe obesity. We expect that this pilot study will provide our team with significant collaboration with telehealth and experience to support a larger randomized controlled trial in an underserved population that includes youth with obesity who are racially diverse and are receiving specialty medical care. An intervention that is feasible and effective, as well as easily accessible through web-based telehealth for diverse and underserved families may be a practical, acceptable, and cost-effective weight management treatment for youth seeking treatment for severe obesity.

## Abbreviations

ASA24: Automated Self-administered 24-hour Recall
RPMS: remote patient monitoring system
UMMC: University of Mississippi Medical Center
